# Comparison of Interactions Between Soy Protein Isolate and Three Folate Molecules: Effect on the Stabilization, Degradation, and Oxidization of Folates and Protein

**DOI:** 10.3390/foods13244033

**Published:** 2024-12-13

**Authors:** Linlin He, Yuqian Yan, Dandan Song, Shuangfeng Li, Yanna Zhao, Zhuang Ding, Zhengping Wang

**Affiliations:** 1 Institute of Biopharmaceutical Research, Liaocheng University, Liaocheng 252059, China; helinlin2022@163.com (L.H.); yanyuqian24@163.com (Y.Y.); songdandan0423@163.com (D.S.); ynzhao2011@163.com (Y.Z.); wangzhengping@lcu.edu.cn (Z.W.); 2 School of Pharmaceutical Science and Food Engineering, Liaocheng University, Liaocheng 252059, China; lishuangfeng202411@163.com

**Keywords:** soy protein, folic acid, L-5-methyltetrahydrofolate, non-covalent interactions, stability, degradation pathway

## Abstract

This study selected three approved folate sources—folic acid (FA), L-5-methyltetrahydrofolate (MTFA), and calcium 5-methyltetrahydrofolate (CMTFA)—to explore their interaction mechanisms with soy protein isolate (SPI) through spectrofluorometric analysis and molecular docking simulations. We investigated how these interactions influence the structural and physicochemical stability of folates and SPI. Three folates spontaneously bound to SPI, forming complexes, resulting in a decrease of approximately 30 kJ·mol^−1^ in Gibbs free energy and an association constant (K_a_) of 10^5^ L·mol^−1^. The thermodynamic parameters and molecular docking study revealed the unique binding mechanisms of FA and MTFA with SPI. FA’s planar pteridine ring and conjugated double bonds facilitate hydrophobic interactions, whereas MTFA’s reduced ring structure and additional polar groups strengthen hydrogen bonding. Although the formation of SPI–folate complexes did not result in substantial alterations to the SPI structure, their binding has the potential to enhance both the physical and thermal stability of the protein by stabilizing its conformation. Notably, compared with free FA, the FA-SPI complexes significantly enhanced FA’s stability, exhibiting 71.1 ± 1.2% stability under light conditions after 9 days and 63.2 ± 2.6% stability in the dark after 60 days. In contrast, no similar effect was observed for MTFA. This discrepancy can be ascribed to the distinct degradation pathways of the Fa and MTFA molecules. This study offers both theoretical and experimental insights into the development of folate-loaded delivery systems utilizing SPI as a matrix.

## 1. Introduction

Owing to their sustainability, environmental friendliness, nutritive value, and health benefits, plant-based proteins have attracted increased interest. They have been rapidly adopted by consumers, and their popularity as excellent substitutes for animal proteins is expected to increase in the coming decade [1]. Soybean protein isolate (SPI) is a leading plant-based protein, and its production methods, structures, properties, and functions have been thoroughly researched [2]. It has demonstrated exceptional characteristics and processability, with a unique taste, along with film-forming, gelling, emulsifying, and foaming properties; water solubility; and biocompatibility [3,4]. Consequently, SPI is extensively utilized as a functional additive in the food and beverage sector.

Folate, or vitamin B_9_, encompasses heterocyclic compounds such as folic acid, tetrahydrofolate, and 5-methyltetrahydrofolate, characterized by a pterin ring, p-aminobenzoic acid, and one or more γ-linked L-glutamic acid residues [5]. This essential nutrient is primarily found in green leafy vegetables and is crucial for human metabolism, supporting normal cell division and growth by facilitating amino acid and nucleotide synthesis [6]. Folic acid (FA), a synthetic oxidized folate, is widely used in food fortification and pharmaceutical formulations due to its ease of production and chemical stability [5]. Recently, L-5-methyltetrahydrofolate (MTFA) has garnered increased attention from researchers, consumers, and food manufacturers. MTFA is a major natural folate; it offers direct utilization benefits without requiring metabolism [7], prevents the masking of vitamin B_12_ deficiency [8], and lowers the risks of cardiovascular disorder in perinatal women [9]. The commercially available form of MTFA is primarily administered as calcium 5-methyltetrahydrofolate (CMTFA). In food applications, CMTFA is frequently utilized as an additive to simultaneously supply folate and calcium. Relative to free MTFA, calcium ions in CMTFA can influence protein structure and aggregation. However, both FA and MTFA/CMTFA are sensitive to oxygen, light, pH, and temperature, presenting challenges for their preservation during food processing and storage.

Protein complexes are efficient carriers, safeguarding bioactive components from degradation and enhancing their bioavailability [4]. By utilizing robust non-covalent interactions, macromolecular proteins can encapsulate small molecules, thereby enhancing their dispersibility and solubility while offering a protective barrier to labile molecules [10,11]. Prior studies on protein-based complexes have predominantly utilized proteins derived from animals, such as whey protein, casein, and gelatin [5]. Plant-based proteins, particularly SPI, offer a viable alternative to animal-derived proteins and have been widely studied as carriers to encapsulate diverse bioactive components, such as poorly soluble β-carotene, curcumin, phytosterols, and vitamin D_3_, as well as soluble saponins, flavonoids, polyphenols, and vitamin B_12_ [2,11]. Ma et al. [12] showed that forming SPI–anthocyanin complexes improves the chemical stability of anthocyanins at 80 °C. Similarly, Chen et al. [13] observed that SPI–curcumin complexes markedly retarded curcumin degradation during extended storage. Yuan et al. [14] showed that SPI complexation decelerates the release and diminishes the gastric degradation of encapsulated piceatannol and oxyresveratrol. These findings collectively indicate that non-covalent interactions with SPI offer substantial protection for labile bioactive compounds. SPI-FA non-covalent complexes have also been reported by Ochnio et al. [15], who showed a strong interaction with a binding constant of 10^4^ M^−1^. Their study assessed the bioavailability of FA using biomass production in a folate-dependent *Lactobacillus casei* strain, BL23. It demonstrated that the SPI-FA complexes significantly enhanced biomass production and concluded that soybean proteins may facilitate the transport of FA into the cell interior.

Using plant-based proteins as protection carriers is a promising strategy for improving the storage stability of labile compounds [2]. Soybean protein isolate (SPI) has garnered significant attention within the food industry due to its natural capacity to encapsulate sensitive nutrients. As crucial food ingredients, folates face challenges pertaining to their instability in aqueous solutions, which impede the development of fortified liquid products with extended shelf lives. The interactions between SPI and folates may mitigate their degradation during food processing by forming stable complexes, preserving their nutritional value under storage or processing conditions. However, the effect of SPI on the chemical stability of folate molecules has not been directly proven. To the best of our knowledge, the interaction mechanisms between SPI and MTFA/CMTFA has not yet been reported. In this study, the interaction mechanisms between SPI and FA/MTFA/CMTFA were investigated through multiple spectroscopic approaches and the molecular docking technique. Subsequently, the chemical stability of FA/MTFA bound to SPI was examined over 3–60 days of storage. The degradation products of folate molecules were analyzed using liquid chromatography–mass spectrometry (LC-MS) to clarify the impact of SPI complexation on their degradation behaviors.

## 2. Materials and Methods

### 2.1. Materials

Folic acid (FA, purity ≥ 97%) was obtained from Macklin, Shanghai, China. 5-methyltetrahydrofolate (MTFA, purity ≥ 98%) and calcium 5-methyltetrahydrofolate (CMTFA, purity ≥ 95%) were sourced from Aladdin, Shanghai, China. The FA and MTFA reference standards (Lot# SF8060 and SL9530) used in the quantitative analysis were obtained from Solarbio (Beijing, China). Soybean protein powder (Lot# 105900) and 5,5′-dithiobis (2-nitrobenzoic acid) (DTNB, purity ≥ 98%) were obtained from Adamas-beta, Shanghai, China. ANS (8-Anilino-1-naphthalenesulfonic acid, purity ≥ 96%) was sourced from Macklin. Chromatography-grade methanol was sourced from Fisher Scientific, Pittsburgh, Pennsylvania, USA. This study employed analytical-grade chemicals, and water was purified via a Milli-Q system (Millipore, Bedford, MA, USA).

### 2.2. Preparation of Soybean Protein Isolate (SPI)

The SPI was prepared with minor modifications following the method outlined by Sui et al. [16]. Soybean protein powder was mixed with water in a 1:8 weight-to-volume ratio. The solution’s pH was set to 8.0 with 2 M NaOH and stirred at room temperature for 1 h. The insoluble components were removed via centrifugation at 9200 RPM (9000× *g*) for 20 min at 4 °C. The supernatant was collected post-centrifugation, and its pH was adjusted to 4.5 with 2 M HCl. The supernatant was stored at 4 °C overnight to ensure complete precipitation of the SPI. Proteins were isolated via centrifugation at 7800 RPM (6500× *g*) for 30 min at 4 °C and then freeze-dried, ground, and stored for later analysis. The protein content of the resulting SPI was quantified using the Kjeldahl method, yielding a value of 83.2 ± 1.0%.

### 2.3. Preparation of SPI–Folate Complexes

SPI–folate complexes were prepared following the method described by Li et al. [17]. Lyophilized SPI powder was accurately weighed and dissolved in 10 mM of phosphate-buffered saline (PBS, pH 7.4) to prepare a 5 mg/mL SPI solution. The solution was stirred at room temperature for 2 h to ensure the complete hydration and dissolution of the SPI powder. Afterward, the solution was transferred to a 277.2 K (4 °C) environment and left to stand for 24 h to further stabilize the protein molecules and ensure full hydration. A 100 μM folate stock solution was freshly prepared by dissolving various folate powders in PBS and stirring at 298.2 K for 30 min until complete dissolution was achieved. The pH of the solution was meticulously monitored and adjusted to 7.4 using 0.1 M sodium hydroxide, as necessary, to ensure consistency with the SPI solution. In subsequent experiments, SPI–folate complexes were formulated by combining the protein and folate stock solutions, followed by dilution with PBS (pH 7.4) to achieve the desired final concentrations. The final concentrations of the SPI–folate complexes are detailed in Section 2.4. The complex samples were mixed in glass tubes and incubated for 30 min at 298.2 K in a light-protected environment. Each complex sample was independently prepared in triplicate for subsequent analysis.

### 2.4. Fluorescence Spectroscopy

Fluorescence measurements were conducted using a Hitachi F-7000 fluorescence spectrophotometer (Hitachi, Tokyo, Japan) equipped with a thermostatic bath. Fluorescence emission spectra were recorded from 300 to 500 nm at 298.2 K, 303.2 K, and 308.2 K, using an excitation wavelength of 280 nm. The scanning speed was maintained at 1200 nm/min, with slit widths for both excitation and emission set at 5 nm. A series of test solutions, comprising 0.5 mg/mL SPI and varying folate concentrations (ranging from 0 to 25 μM), were prepared as detailed in Section 2.3. The working concentrations were determined based on the report by Yuan et al. [14]. To evaluate the effect of calcium ions (Ca^2+^) on the SPI-MTFA complexes, calcium chloride (CaCl_2_) was added as an additional solute to simulate a CMTFA application scenario. To avoid interference from Ca^2+^ concentration fluctuations, Ca^2+^ was consistently maintained at 25 μM, matching the maximum MTFA concentration used in fluorescence analysis. The fluorescence data were corrected using Equation (1), as outlined by Li et al. [18], to remove the inner filter effect:

F_corr_ = F_obs_ × e^(Aem+Aex)/2^ (1)

where F_corr_ and F_obs_ are the corrected and observed fluorescence intensities, respectively. Aex and Aem represent the absorption of the SPI–ligand complex at the excitation and emission wavelengths, respectively.

### 2.5. Surface Hydrophobicity (H_0_) Measurement

The surface hydrophobicity (H_0_) of the SPI, both with and without FA or MTFA, was evaluated using an ANS fluorescence probe [19]. The SPI stock solution was diluted using PBS to obtain concentrations between 0 and 1.5 mg/mL. The protein solution was then mixed with an equal volume of 50 μM folate stock solution. The SPI–folate complexes (3 mL) were incubated with 30 μL of 8 mM ANS solution in PBS at 298.2 K for 30 min, followed by an additional 30 min incubation at the same temperature. The samples’ fluorescence intensity was measured using an F-7000 spectrophotometer, with excitation and emission wavelengths set at 390 nm and 470 nm, respectively. A linearity curve was created by plotting the protein concentration on the x-axis against fluorescence intensity on the y-axis. The slope of this curve was determined and utilized as a measured value of the protein’s H_0_.

### 2.6. FTIR Spectroscopy

Prior to FTIR spectroscopy analysis, complex samples comprising 2.5 mg/mL SPI and 10 µM folates were prepared and subsequently freeze-dried following the method of Zhang et al. [20]. The resulting dried SPI–folate complexes (1 mg) were then combined with potassium bromide (KBr, 100 mg) and compressed into tablet form. FTIR spectra for SPI–folate complexes were obtained using a Nicolet iS50 FTIR spectrometer (Thermo Fisher Scientific, San Jose, CA, USA), covering a spectral range of 400–4000 cm^−1^. The 1600–1700 cm^−1^ amide I band spectra were analyzed with the PeakFit software, version 4.12 (Thermo Fisher Scientific), to assess the protein’s secondary structure content.

### 2.7. Molecular Docking

β-conglycinin (7S globulins) and glycinin (11S globulins) accounted for over 70% of the total SPI content [21]. Consequently, these two proteins were chosen as representative receptors for the molecular docking simulations involving SPI and folate ligands. Given that CMTFA exists in solution as MTFA, FA, and MTFA, they were chosen as ligands for this study. The 3D structures of 7S (PDB ID: 3AUP) and 11S globulins (PDB ID: 1OD5) were obtained from the Protein Data Bank (https://www.rcsb.org, accessed on 15 September 2024), and hydrogen atoms were added using AutoDock Tools (ADT3). The three-dimensional structures of FA (PubChem CID 135398658) and MTFA (PubChem CID 13539856) were obtained from the PubChem database (https://pubchem.ncbi.nlm.nih.gov, accessed on 15 September 2024). These structures were subsequently processed using ADT3 to eliminate water molecules and incorporate hydrogen atoms. Docking grids were generated utilizing AutoGrid 8.9, and molecular docking was performed with AutoDock Vina 1.2.0 (Scripps Research, La Jolla, CA, USA). The grid box dimensions of the 7S and 11S globulins used for molecular docking were 60 × 61 × 67 and 53 × 90 × 47 (x × y × z), with 0.375 Å of spacing. The optimal conformation was selected based on the lowest binding energy criterion for interaction analysis. The results were then visualized and analyzed using PyMOL version 3.1 (Schrödinger, Inc., New York, NY, USA).

### 2.8. Differential Scanning Calorimetry (DSC)

The thermal properties of the SPI and SPI–folate complexes were analyzed utilizing a Q2000 differential scanning calorimeter (TA Instruments, New Castle, DE, USA). Lyophilized powders of SPI and SPI–folate complexes (5 mg each) were sealed in aluminum pans and heated from 25 °C to 200 °C at 10 °C/min under a 50 mL/min nitrogen flow to observe heat flow changes. An empty aluminum pan was used as the reference.

### 2.9. Storage Stability

#### 2.9.1. Sample Storage

The physical stability of the SPI–folate complexes and the chemical stability of folate molecules were assessed under light and dark conditions, respectively. Both the SPI–folate complexes and unprotected folate solutions, each measuring 10 mL, were placed in 20 mL sealed glass vials. The folate concentration in the samples was maintained at 10 μM, while the SPI concentration was set at 2.5 mg/mL. To inhibit microbial growth, sodium azide was added to all samples at a concentration of 0.01 wt%. The samples were incubated at 25 °C in a Binder GmbH MKF720 climate chamber (Binder GmbH, Tuttlingen, Germany). Samples were exposed to 200-lux incandescent light from a built-in lamp positioned 30 cm away. The samples containing FA were exposed for 9 days, while those containing MTFA were exposed for 3 days. Under dark conditions, the FA-containing samples were assessed over 60 days, and the MTFA-containing samples were evaluated for 7 days. Unprotected folate solutions served as the blank control in this experiment.

#### 2.9.2. Physical Stability

The Zetasizer Nano ZS system (Malvern Instruments, Malvern, UK) was used to quantitatively assess the average particle size and zeta potential of the complexes before and after storage, evaluating their physical stability. The samples remained undiluted and unfiltered throughout the measurement process.

#### 2.9.3. Chemical Stability

The SPI–folate complexes and folate solution samples were collected at predetermined intervals for analysis. FA and MTFA in the samples were quantitatively analyzed using a Shimadzu LC-MS 8040 system with a C18 analytical column (QuikSep SP ODS-AQ, 5 μm, 4.6 mm × 250 mm, H&E Technology, Beijing, China) at 30 °C. Standard curves for folate content (1–500 μM, R2 > 0.9998) were generated using FA and MTFA reference standards. Molecular weight analysis was performed using positive electrospray ionization mode with the following conditions: 3.0 L/min of spray gas flow, 15.0 L/min of drying gas flow, and 400 °C drying temperature. The detailed chromatographic conditions are described in a study by Zhang et al. [20]. The retention rate (RS) of folate molecules was determined using Equation (2), as referenced in [22]:

RS (%) = C_s_/C_0_ (2)

where C_s_ represents the concentration of residual folate molecules (μM), and C_0_ denotes the initial folate concentration (10 μM).

### 2.10. Sulfhydryl Analysis

The Ellman spectrophotometric method [16] was employed to quantify the total sulfhydryl content in both the SPI and SPI–folate complexes. The SPI and folate concentrations were 2.5 mg/mL and 10 μM, respectively. The Ellman reagent was formulated by dissolving 4 mg of DTNB in 1 mL of triglycine buffer (86 mM Tris, 90 mM glycine, 4 mM Na_2_EDTA, pH 8.0). A 50 μL aliquot of the Ellman reagent was added to 3 mL of the sample and incubated at 25 °C for 30 min in the dark, using PBS as the blank. The sulfhydryl contents (SH) were determined using Equation (3).

SH (μmol/g) = 73.53 × A_412_/C (3)

### 2.11. Statistical Analysis

All experiments were conducted in triplicate, and the results are expressed as the mean ± standard deviation. Statistical analyses were conducted using one-way ANOVA and significance tests with SPSS 26.0 (SPSS Inc., Chicago, IL, USA). Significant differences were assessed using a *t*-test, with a *p*-value of < 0.05 indicating statistical significance.

## 3. Results and Discussion

### 3.1. Interaction Mechanisms Between SPI and Folates

#### 3.1.1. Analysis of Fluorescence Spectroscopy

**Figure 1:**
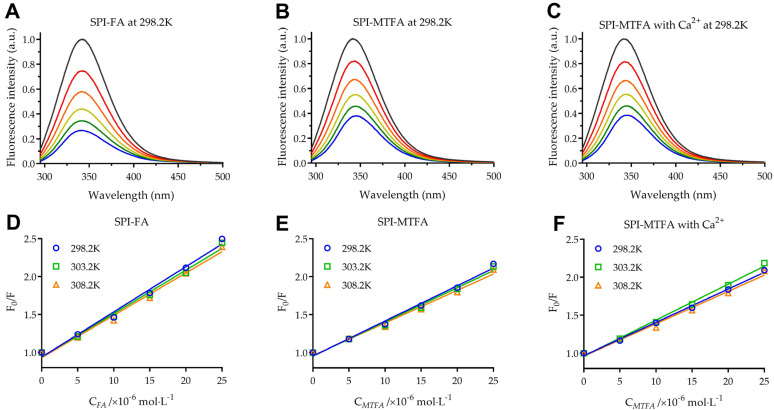
Fluorescence emission spectra of 5 g/L soy protein isolation (SPI) in the presence of folic acid (FA), (**A**) L-5-methyltetrahydrofolate (MTFA), (**B**) and MTFA with 25 μM calcium ion (Ca^2+^) (**C**) at different concentrations ranging from 0 to 25 μM (T = 298.2 K, pH = 7.4, λex = 280 nm). (**D**–**F**) Stern–Volmer plots of the interactions between SPI and three folate molecules at 298.2–308.2 K.

Fluorescence spectroscopy is an effective and practical method for detecting protein–ligand complexes and assessing their interactions owing to its high sensitivity and non-invasive nature. The impact of folate molecules on the intrinsic fluorescence spectrum of SPI was investigated to elucidate the binding interaction between SPI and folates. Figure 1 illustrates the fluorescence spectra of SPI solutions with varying concentrations (0–25 μM) of folate molecules (FA, MTFA, and CMTFA) at 298.2 K. The peak fluorescence intensity of SPI was noted at around 342 nm, aligning with Yuan et al. [14], who investigated its fluorescence spectrum to explore its binding interactions with piceatannol and oxyresveratrol. Figure 1A–C show that increasing folate concentrations progressively decreased the SPI fluorescence intensity, indicating that folate interaction causes SPI fluorescence quenching. This indicates that folate molecules were successfully conjugated with SPI and that increased folate molecule concentrations promoted the formation of SPI–folate complexes. The fluorescence intensity of SPI decreased by 73.4% with 25 μM FA, 62.4%, and 62.2% with 25 μM MTFA and MTFA with Ca^2+^, respectively. The results indicate that FA demonstrates a higher binding affinity to SPI than MTFA, possibly because of their structural differences. The hydrophobic aromatic ring in FA potentially establishes stronger hydrophobic interactions with the hydrophobic amino acid residues in SPI. Conversely, the increased presence of secondary amine groups in the hydrogenated pteridine ring of MTFA diminishes its hydrophobicity, reducing its binding affinity with SPI. Furthermore, the results suggest that calcium ions (Ca^2+^) do not influence the interaction between MTFA and SPI. The maximum emission wavelength of SPI showed a minor red shift from 341.8 nm to 342.8 nm with an increase in the FA concentration from 0 to 25 μM. The maximum emission wavelength of SPI shifted from 342.6 nm to 346.2 nm when exposed to 25 μM of MTFA, both with and without Ca^2+^. The observed red shift in the maximum emission wavelength suggests that the binding of FA and MTFA appears to expose tryptophan (Trp) and tyrosine (Tyr) residues to a more hydrophilic environment [14].

The Stern–Volmer equation, Equation (4), was used to calculate the quenching parameters by substituting the fluorescence intensity of SPI at different folate concentrations (0–25 μM) [23]. In this equation, F_0_ and F represent the fluorescence intensities of SPI without and with the quencher (folates), respectively. Reference [Q] is the concentration of the quencher; K_q_ denotes the bimolecular quenching rate constant, and τ_0_ is the fluorophore’s average lifetime without the quencher, valued at 10^−8^ s.

F_0_/F = 1 + *K*_q_·τ_0_·[Q] (4)

Figure 1D–F demonstrate a strong linear relationship between F_0_/F and quencher concentration, [Q], at temperatures of 298.2 K, 303.2 K, and 308.2 K. Ranging from 4.27 to 5.97 × 10^12^ L·mol^−1^·s^−1^, the K_q_ values in Table 1 far surpass the biomolecular collision quenching constant of 2 × 10^10^ L·mol^−1^·s^−1^, indicating a static quenching mechanism [24]. The results suggest that SPI and folate molecules form a ground-state complex with a strong binding affinity.

#### 3.1.2. Binding Constants and Binding Force

For the static quenching mechanism, the association constant (K_a_) between biomacromolecules and ligands can be determined using the Lineweaver–Burk equation, Equation (5) [25].

Log (F_0_/F − 1) = log*K*_a_ + n·log[Q] (5)

The association constant (K_a_) and binding site number (n) are presented in Table 1. The n values obtained at three different temperatures were approximately one, suggesting that folate molecules interact with a singular binding site on SPI. The K_a_ value of the SPI–FA system was higher than that of the SPI–MTFA system, indicating a stronger binding affinity between FA and SPI than between MTFA and SPI. This observation aligns with the more pronounced quenching effect of FA on SPI fluorescence. The K_a_ value for the SPI-FA system rose from 2.72 to 5.35 × 10^5^ L·mol^−1^ as the temperature increased from 298.2 K to 308.2 K. These findings align with the results reported by Ochnio et al. [15], who reported K_a_ values of 2.40 × 10^4^ M^−1^ and 1.87 × 10^5^ M^−1^ for the binding of FA with SPI 7S and 11S globulins at 298.2 K, respectively. Our findings suggest that the binding affinity between SPI and FA improves with increasing temperature. A similar observation was reported by Cen et al. [26], who demonstrated that the K_a_ of the ovalbumin–FA complex rises with elevated preparation temperature, primarily due to intensified hydrophobic interactions. In contrast, the K_a_ of the MTFA-SPI system decreased from 2.70 to 1.45 × 10^5^ L·mol^−1^ as the temperature increased, suggesting that the interaction mechanisms of MTFA with SPI differs from that of FA. Furthermore, in the presence of Ca^2+^, the K_a_ value of the SPI-MTFA system varied from 2.69 to 1.22 × 10^5^ L·mol^−1^ over a temperature range of 298.2–308.2 K, suggesting that low concentrations of Ca^2+^ do not influence the binding of MTFA to SPI.

The interaction mechanisms between SPI and folates were clarified by deriving the thermodynamic parameters using the Van’t Hoff and Gibbs–Helmholtz equations. In this context, ∆G° is the Gibbs free energy change; ∆H° is the enthalpy change; ∆S° is the entropy change; R represents the universal gas constant (8.314 J·mol^−1^·K^−1^), and T denotes the thermodynamic temperature.

ln*K*_a_ = − ΔH/(R·T) + ΔS/R (6)

ΔG = ΔH − T·ΔS = − R·T·ln*K*_a_ (7)

The matched Van’t Hoff plots for the binding interaction between the SPI and folates demonstrated strong linearity (R^2^ > 0.98, Figure S1), and the corresponding thermodynamic parameters are illustrated in Table 1. The ΔG° values are uniformly negative, signifying that the binding process between SPI and folates occurs spontaneously. For the SPI-FA system, the enthalpy change (ΔH°) is 51.73 kJ·mol^−1^, and the entropy change (ΔS°) is 277.65 J·mol^−1^·K^−1^. The positive ΔH° and ΔS° values suggest that hydrophobic interactions primarily drive the endothermic formation of the SPI-FA complex. This finding aligns with previous research by Teng et al. [27], emphasizing the crucial impact of hydrophobic interactions in forming FA-SPI nanoparticles. In the SPI-MTFA system, the enthalpy change (ΔH°) is −60.13 kJ·mol^−1^, and the entropy change (ΔS°) is −97.44 kJ·mol^−1^. The negative value of ΔH° indicates that the interaction between SPI and MTFA is exothermic, while the negative value of ΔS° suggests a decrease in the flexibility of the resulting complex. This observation implies that SPI-MTFA complexes adopt a more rigid conformation, which is generally considered beneficial for maintaining the stability of the complex system and encapsulating labile compounds. The negative values of ΔH° and ΔS° suggest that van der Waals forces and hydrogen bonding predominantly govern interactions within the SPI-MTFA complex [23]. These interactions may diminish at elevated temperatures, implying that moderate heating could decrease the stability of SPI-MTFA complexes, facilitating the release of MTFA to enhance nutritional quality for food applications [14]. Unlike FA, the molecular configuration of MTFA incorporates a hydrogenated pteridine ring. This structural alteration reduces the hydrophobicity of MTFA and introduces additional amino groups, providing increased potential sites for hydrogen-bonding interactions between MTFA and SPI. The SPI-MTFA complex displays comparable thermodynamic parameters with or without 25 μM Ca^2+^, indicating that Ca^2+^ does not notably affect the binding affinity between MTFA and SPI.

### 3.2. Surface Hydrophobicity

**Figure 2:**
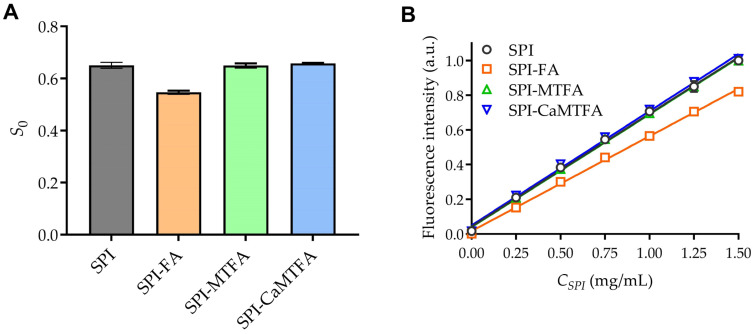
(**A**) Surface hydrophobicity (H_0_) of soy protein isolation (SPI) and SPI–folate complexes. (**B**) Linearity curve plotting the relative fluorescence intensity of the ANS probe with concentrations from 0 to 1.5 g/L of the SPI and SPI–folate complexes. FA, MTFA, and CMTFA denote folic acid, L-5-methyltetrahydrofolate, and calcium L-5-methyltetrahydrofolate, respectively.

The surface hydrophobicity (H_0_) of SPI, with and without folate molecules, was evaluated using an ANS hydrophobic fluorescence probe (Figure 2) [28]. The formation of the SPI-FA complexes resulted in a 15.94% reduction in H_0_, suggesting that FA occupies hydrophobic regions on the SPI surface, thereby decreasing the available binding sites for ANS. Tong et al. [29] reported a similar phenomenon, where epigallocatechin gallate (EGCG) interacted with SPI fibrils through hydrophobic interactions, reducing protein H_0_. Nevertheless, incorporating MTFA/CMTFA did not exert a statistically significant impact on the H_0_ of SPI (*p* > 0.05), indicating that MTFA does not obscure the hydrophobic regions accessible for ANS binding on the SPI surface. These findings imply that FA interacts with the SPI surface primarily through hydrophobic forces, whereas hydrophobic interactions may not constitute the primary driving force governing the interaction between SPI and MTFA, a conclusion corroborated by the fluorescence analysis.

### 3.3. Fourier Transform Infrared (FTIR) Spectra

**Figure 3:**
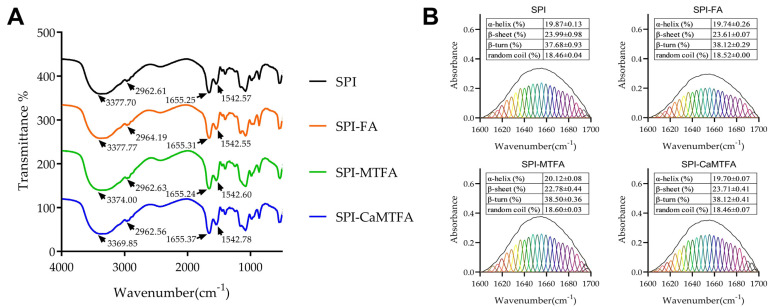
(**A**) Fourier transform infrared (FTIR) spectra and (**B**) curve-fitted analysis of the amide I region (1600–1700 cm^−1^) for soy protein isolate (SPI) and SPI–folate complexes. The table in (**B**) summarizes protein secondary structure contents derived from integrating the area under each band in the curve-fitting analysis.

The FTIR spectra of the SPI and SPI–folate complexes were obtained within the 400–4000 cm^−1^ range, as illustrated in Figure 3. The broad peak at 3000–3700 cm^−1^ is linked to the stretching vibrations of −OH and −NH groups in SPI. Notably, this peak exhibits a red shift from 3377.70 cm^−1^ to 3374.00 cm^−1^ and 3369.85 cm^−1^ in the MTFA-SPI and CMTFA-SPI complexes, respectively. This shift suggests the formation of significant hydrogen-bonding interactions during the binding of SPI with MTFA/CMTFA. In contrast, such a shift is absent in the SPI-FA complexes, indicating a weaker hydrogen bonding interaction between SPI and FA. The absorption peak at 2962.61 cm^−1^ corresponds to the C-H stretching vibration of methyl and methylene groups in the protein, and its shift can be used to detect hydrophobic interactions between proteins and ligands [30]. Unlike SPI, the peak of the SPI-FA complex shows a slight red shift to 2964.19 cm^−1^, possibly indicating hydrophobic interactions between SPI and FA during complex formation. In contrast, no such shift was observed for the SPI-MTFA complex, consistent with the results of the fluorescence and hydrophobicity tests. The amide I (1700–1600 cm^−1^) and amide II (1600–1500 cm^−1^) bands—linked to C = O stretching and C-N stretching/N-H bending vibrations, respectively—indicate protein structure [31]. Figure 3 shows that folate molecules do not significantly alter the amide I and II bands. The Fourier self-deconvolution technique was applied to the amide I band for qualitative insights into SPI’s secondary structure (Figure 3B). The secondary structure composition of SPI consisted of 19.87% α-helices, 23.99% β-sheets, 37.68% β-turns, and 18.46% random coils, with a notably higher proportion of β-turns. The addition of folates did not alter this proportion in the secondary structure (*p* < 0.05), suggesting that the interaction with folates does not significantly affect the SPI conformation. This observation is consistent with the study by Chen et al. [32], which reported no significant alterations in the secondary structure of SPI upon binding with curcumin.

### 3.4. Molecular Docking Study

**Figure 4:**
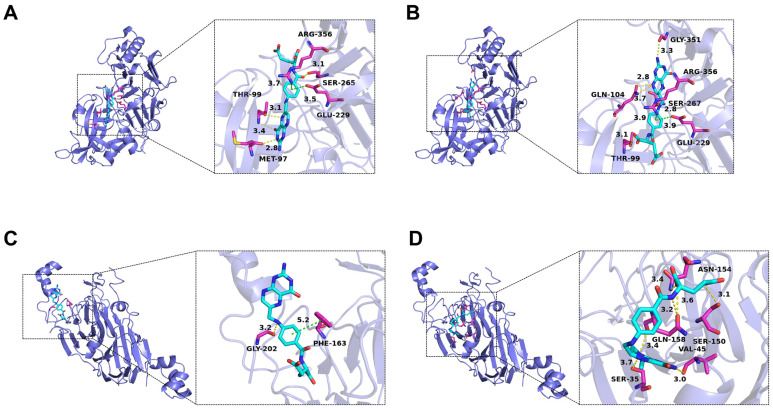
Binding model diagram of folic acid (FA) and L-5-methyltetrahydrofolate (MTFA) to soy β-conglycinin (7S globulins) (**A**,**C**) and glycinin (11S globulins) (**B**,**D**). The left and right images show the binding site and the interactions between folates and surrounding amino acid residues, respectively.

Molecular docking was utilized to predict the binding positions and affinities between 7S/11S globulins and FA/MTFA, visually elucidating the interaction mechanisms between SPI and folates (Figure 4). For 7S globulins, the binding positions of FA and MTFA were similar; however, the specific amino acids involved and the relative interaction modes exhibited significant differences. The central benzene ring of FA engaged in strong hydrophobic interactions with Glu229 and Arg356, exhibiting interaction distances of 3.5 Å and 3.7 Å, respectively. The pteridine ring of FA formed three hydrogen bonds with Met97 and Thr99, with bond lengths of 2.8 Å, 3.1 Å, and 3.4 Å. In contrast, the pteridine ring of MTFA engaged in hydrogen bonding with more amino acid residues, namely, Thr99, Gln104, Ser267, and Gly351, with bond lengths between 2.8 Å and 3.7 Å. Furthermore, the benzene ring of MTFA engaged in hydrophobic interactions with Glu229 and Arg356, maintaining an interaction distance of 3.9 Å. The binding sites and interaction modalities of FA and MTFA on the surface of 11S globulins demonstrated more pronounced differences. The central benzene ring of FA interacts hydrophobically with Phe163 at 5.2 Å, and its secondary amine group forms a hydrogen bond with Gly202 at 3.2 Å. The pteridine ring and glutamic acid moiety of MTFA formed multiple hydrogen bonds with amino acid residues such as Ser35, Val45, Ser150, Asn154, and Gln158, with bond lengths between 3.0 and 3.7 Å. Notably, no hydrophobic interactions between 11S globulins and MTFA were observed. The molecular docking analysis indicated that significant hydrophobic interactions were involved in the binding between SPI and FA, whereas hydrogen-bonding interactions predominantly facilitated the formation of SPI-MTFA complexes. These observations aligned with the fluorescence spectroscopy findings. The binding energies for FA with 7S and 11S globulins were −35.15 kJ/mol and −29.71 kJ/mol, respectively, whereas for MTFA, they were −37.24 kJ/mol and −30.96 kJ/mol. These findings corroborate the experimental data obtained from fluorescence spectroscopy, further substantiating the spontaneous binding of SPI and folates. Importantly, in addition to 7S and 11S globulins, SPI comprises other protein constituents, including lipophilic proteins and lunasin peptide [33], which may also engage in non-covalent interactions with folate molecules. In our study, selecting 7S and 11S globulins as representative proteins for molecular docking experiments is justified by their predominance in SPI, constituting over 70% of its total mass. The fluorescence spectroscopy analysis and molecular docking study reveal a strong interaction between folate molecules and SPI. The formation of protein-binding complexes emerges as a potential strategy for enhancing the stability of small-molecule ligands while preserving the nutritional value of fortified foods. Consequently, the stability of SPI–folate complexes and their impact on the chemical stability of folates were further assessed.

### 3.5. Thermal Stability of SPI–Folate Complexes

**Figure 5:**
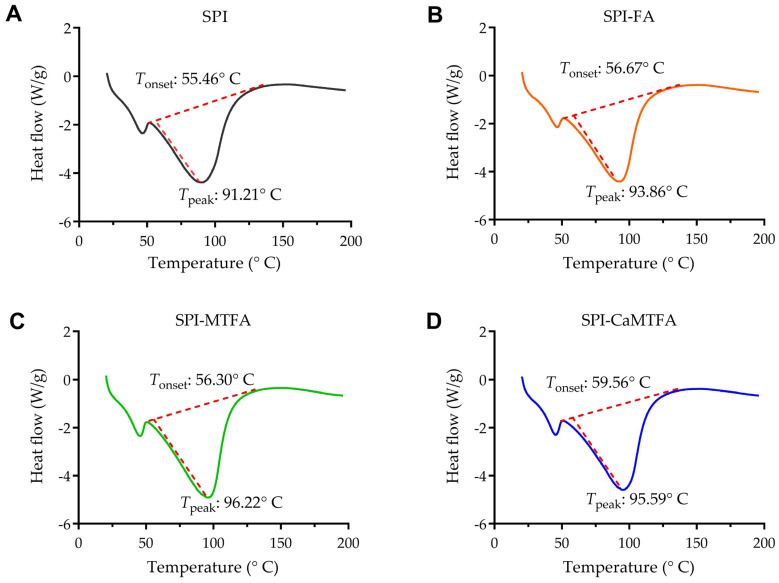
Differential scanning calorimetry (DSC) thermograms of soy protein isolation (SPI) (**A**) and three SPI–folate complexes (**B**–**D**).

Differential scanning calorimetry (DSC) is used as a thermal analysis method to evaluate phase transitions in biomaterials [34]. The thermal characteristics of the SPI and SPI–folate complexes are illustrated in Figure 5. The initial denaturation temperature (T_onset_) and maximum degradation temperature (T_peak_) measured via DSC indicate the structural stability of the biomacromolecule or biopolymer being studied [35]. All samples exhibited a minor endothermic peak at 50–60 °C due to water evaporation. In the DSC thermogram of the SPI sample, T_onset_ and T_peak_ were recorded at 55.46 °C and 91.21 °C, respectively, aligning with the values reported by Rawel et al. [28]. Upon interaction with folates, the endothermic T_onset_ values of SPI-FA, SPI-MTFA, and SPI-CMTFA shifted to 56.67 °C, 56.30 °C, and 59.56 °C, respectively. Correspondingly, their T_peak_ values shifted to 93.86 °C, 96.22 °C, and 95.59 °C, respectively. The formation of SPI–folate complexes appears to enhance the thermal stability of SPI. The SPI-CMTFA exhibits slightly improved thermal stability compared with MTFA and FA, possibly owing to the presence of Ca^2+^. Permyakov and Berliner [36] found that the binding of Ca^2+^ to α-lactalbumin (α-LA) substantially enhances its thermal stability, increasing the thermal transition temperature by over 40 °C. Wang et al. [37] demonstrated that the complexation of divalent metal ions with the hydroxyl groups of the protein enhances the stability of the complex conformation.

### 3.6. Physical Stability of SPI–Folate Complexes

Table 2 presents an evaluation of the SPI–folate complexes’ physical stability through particle size and ζ-potential measurements. The initial average particle size of SPI was 30.6 ± 1.1 nm, consistent with the 30 ± 4 nm range reported by Ren et al. [38]. The particle sizes for the freshly prepared SPI-FA, SPI-MTFA, and SPI-CMTFA complexes were 30.3 ± 0.9 nm, 30.1 ± 0.6 nm, and 30.4 ± 0.3 nm, respectively. These findings suggest that folate incorporation did not significantly alter or aggregate the protein particles. This phenomenon may be due to the relatively low concentration of folate molecules and their non-covalent interactions with SPI. In a previous study on SPI-FA complexes, Ochnio et al. [15] reported that high concentrations of folic acid (4.5–7.0 mM) lead to SPI aggregation (3.0–5.5 mM). Our study indicates that a 10 μM folate concentration does not modify the SPI particle structure or trigger its aggregation. Additionally, Chen and Xi [39] observed that the non-covalent binding of small-molecular ligands to SPI minimally influences the protein’s particle size distribution, whereas covalent binding significantly affects it. Table 2 indicates that the ζ-potential of native SPI was −10.3 ± 0.2 mV, corroborating the findings of Guo et al. [40]. Unlike SPI, the ζ-potential of the SPI–folate complexes did not demonstrate significant changes, corroborating the results of Ochnio et al. [15], who observed that the ζ-potential of 7S and 11S globulins remained largely stable despite increasing FA concentrations. These results also suggest that the binding between SPI and folates is not driven by electrostatic interaction.

Over 9 days of light exposure, the SPI particle size increased from 30.6 nm to 61.9 nm. This increase can likely be attributed to protein aggregation induced by light exposure. Espinoza and Mercado-Uribe [41] observed similar effects with visible light radiation on β-L-crystallin and ovalbumin proteins. Light exposure can oxidate sulfhydryl groups (−SH) within SPI, facilitating the formation of disulfide bonds (S-S) and consequently inducing cross-linking between protein molecules. This structural alteration exposes hydrophobic regions, enhancing intermolecular interactions and promoting protein aggregation [42]. After 9 days of light exposure, the particle sizes of the SPI-FA complexes measured 55.66 ± 2.83 nm, possibly due to partial protein degradation caused by FA and its photodegradation products under light exposure conditions. Liang et al. [43] showed that milk proteins interact with FA and its photodegradation products, leading to protein unfolding or degradation. During a 60-day storage period in darkness, the SPI particle size increased at a slower rate, reaching 44.96 ± 1.90 nm, whereas SPI–folate complexes maintained a size of about 30 nm. This observation indicates that SPI and its ligand-binding complexes exhibit greater structural stability in darkness than in light. The ζ-potential of SPI and SPI-FA/MTFA complexes showed a notable increase in the magnitude of their negative ζ-potential under both light and dark conditions. The accumulation of degradation products from SPI and folate molecules during storage may explain this phenomenon. The resultant increase in surface charge density enhanced electrostatic repulsion within the system, decelerating the aggregation process. Moreover, the physical state of SPI-CMTFA under various storage conditions was comparable to that of SPI-MTFA. This suggests that a low calcium ion concentration (10 μM) does not influence the physical properties of SPI or its binding interactions with small-molecule ligands.

### 3.7. Chemical Stability of Folates

**Figure 6:**
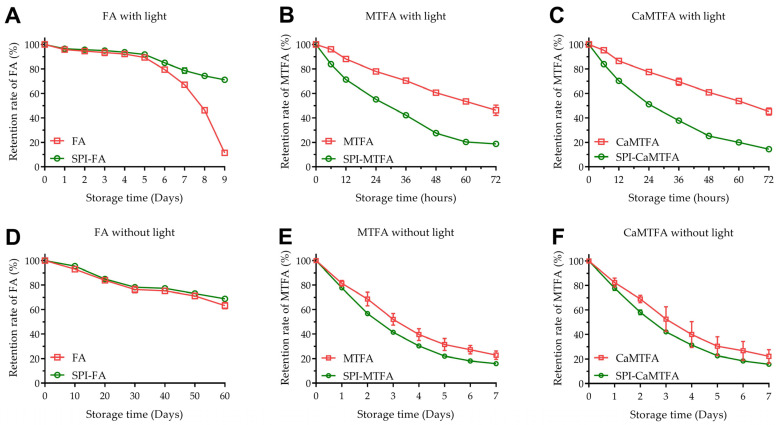
Stability of folic acid (FA) (**A**,**D**), L-5-methyltetrahydrofolate (MTFA) (**B**,**E**), and calcium L-5-methyltetrahydrofolate (CMTFA) (**C**,**F**) in the absence and presence of soy protein isolation (SPI) under light and dark conditions at 25 °C.

Figure 6 illustrates the retention rates of various folate molecules (FA, MTFA, and CMTFA) with and without SPI across two storage conditions. Following 9 days of light exposure, the retention rate of FA significantly declined to 11.3 ± 0.2%. As a photosensitive compound, FA is prone to degradation, resulting in various lower-molecular-weight degradation products due to the generation of singlet oxygen by various forms of optical radiation [44]. The degradation products of FA, including 6-formyltetrahydropterin and pterin-6-carboxylic acid, have been shown to accelerate the photodegradation of FA [45]. The degradation rate of FA increased significantly in the later stages of light exposure compared with the earlier stages (Figure 6). Conversely, when FA formed a complex with SPI, it demonstrated enhanced photostability under identical light conditions, achieving a retention rate of 71.1 ± 1.2% after 9 days. This indicates that SPI can effectively delay the photodegradation of FA. Under conditions devoid of light, FA demonstrated enhanced chemical stability, exhibiting a retention rate of 63.2 ± 2.6% after 60 days of storage. Incorporating SPI further augmented FA stability, achieving a retention rate of 68.8 ± 1.0%. Proteins such as human and bovine serum albumin, β-lactoglobulin, and α-lactalbumin have been shown to partially prevent FA photodegradation [46].

**Figure 7:**
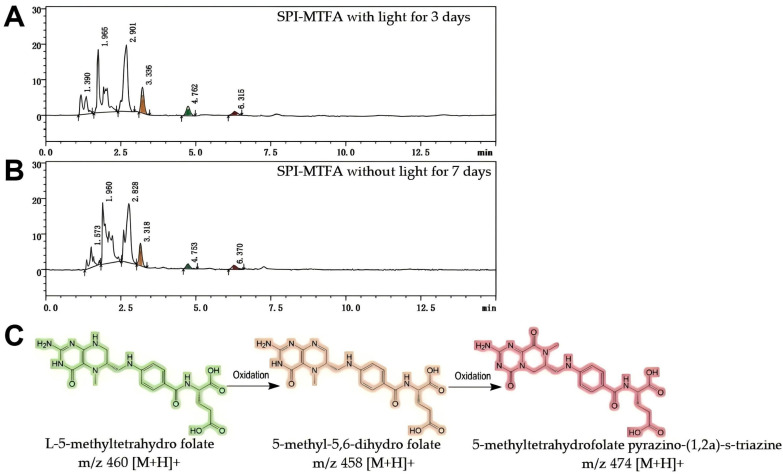
Liquid chromatography chromatograms of the soy protein isolation (SPI)-L-5-methyltetrahydrofolate (MTFA) complexes after 3 days of light (**A**) and 7 days of dark storage (**B**) at 25 °C. (**C**) Degradation products of MTFA during storage and their corresponding *m/z* values determined via mass spectrometry.

MTFA/CMTFA exhibited faster degradation than FA in both light and dark conditions. After 3 days of light exposure, the retention rate of MTFA in control samples dropped to 46.2 ± 4.2%. Unexpectedly, the presence of SPI accelerated the degradation of MTFA with only 18.8 ± 1.4% retention rates after 3 days of light exposure. Similarly, after 7 days of storage in dark conditions, the degradation rate of MTFA in the complexes was higher than in the control sample (16.0 ± 0.5% vs. 22.7 ± 3.4%). To explain this phenomenon, the derivatives of MTFA formed in the storage process were determined via LC-MS (Figures S2 and S3). Unlike the initial SPI-MTFA complexes, the two newly produced compounds were determined after 3 days of light exposure or 7 days of dark storage. Their retention times on the chromatogram were 3.4 and 6.5 min, corresponding to molecular weights of 457 (458 *m/z*, [M+H]^+^) and 473 Dalton (474 *m/z*, [M+H]^+^) (Figure 7). This showed that during both dark and light exposure storage processes, oxidizing MTFA mainly generated 5-methyl-5,6-dihydrofolate (Mw = 457, OXP-I) and 5-methyltetrahydrofolate pyrazino-(1,2a)-s-triazine (Mw = 473, OXP-II) rather than fracturing it into many low-molecular-weight structural fragments [47]. Yin et al. [48] demonstrated that SPI contains higher hydroperoxide and free radical levels than whey protein. Consequently, SPI degraded resveratrol instead of offering a protective effect similar to whey protein, indicating that the pronounced pro-oxidative properties of SPI predispose oxidation-sensitive compounds to oxidation degradation pathways [34]. In contrast to the cleavage degradation observed with FA, MTFA—characterized by a reduced pteridine ring—is more susceptible to entering oxidation degradation pathways. This distinction accounts for the differential impact of SPI on the stability of small molecules. Our prior research demonstrated that forming caseinate–folate complexes effectively delays MTFA degradation. This study indicates that the balance between the antioxidant and pro-oxidative properties of the protein carrier primarily influences its protective effect on oxygen-sensitive components [34]. In addition, CMTFA displayed a similar degradation behavior with MTFA under both conditions (Figures S4 and S5), indicating that adding a small amount of Ca^2+^ does not affect the interaction between SPI and MTFA and their coexistence states.

### 3.8. Sulfhydryl Groups of SPI

**Figure 8:**
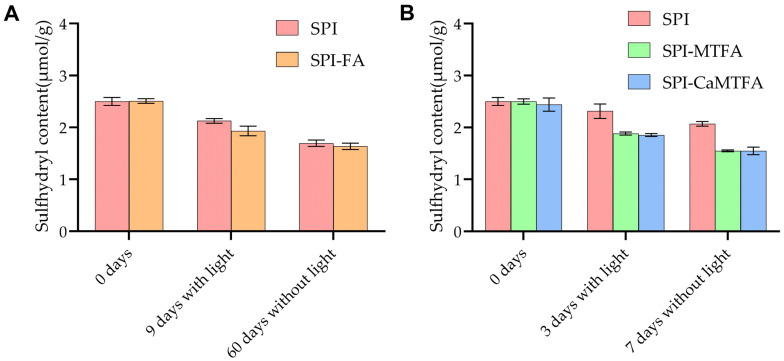
(**A**) Variation in sulfhydryl content of SPI and SPI-FA complexes over 9 days under light exposure and 60 days under dark storage conditions. (**B**) Variation in sulfhydryl content of SPI and SPI-MTFA/CMTFA complexes over 3 days under light exposure and 7 days under dark storage conditions.

Protein oxidation during storage was evaluated by quantifying the sulfhydryl (−SH) group contents in SPI and SPI–folate complexes (Figure 8). The initial free −SH content of SPI was measured at 2.50 ± 0.08 μmol/g, suggesting a relatively high initial oxidation state, potentially attributable to lipoxygenase-mediated lipid oxidation occurring during the extraction process [49]. The −SH content in newly formed SPI–folate complexes was similar to that of SPI alone, suggesting that complex formation did not notably affect the initial −SH levels.

Upon exposure to light for 9 days, the −SH content of SPI further diminished to 2.13 ± 0.04 μmol/g, indicating ongoing protein oxidation. In contrast, the SPI-FA complex more rapidly declined in −SH content, reaching 1.93 ± 0.09 μmol/g. This accelerated oxidation in the SPI-FA complex can be attributed to the additional oxidative effects exerted by the photodecomposition products of folate on SPI. Fu et al. [50] demonstrated that the decomposition of FA sensitizes whey protein isolate to indirect oxidation, resulting in protein unfolding, oligomerization, and degradation. After 60 days of storage under dark conditions, the −SH content of SPI decreased to 1.70 ± 0.06 μmol/g, which was attributed to the extended duration of the oxidation process. The −SH content of the SPI-FA complex remained stable at 1.64 ± 0.06 μmol/g, unlike SPI alone. This stability can be attributed to the relative stability of FA in the absence of light, which prevents the rapid accumulation of small-molecular photodecomposition products. These findings align with the outcomes of chemical stability experiments.

After 3 days of light exposure, the −SH content of SPI decreased to 2.31 ± 0.14 μmol/g and further reduced to 2.07 ± 0.04 μmol/g following 7 days in darkness. The −SH content in SPI-MTFA/CMTFA complexes decreased swiftly to 1.55 ± 0.02/1.55 ± 0.07 μmol/g under light exposure and 1.88 ± 0.03/1.85 ± 0.03 μmol/g in darkness. These findings suggest that MTFA/CMTFA significantly enhances SPI oxidation, exhibiting a stronger pro-oxidant effect on the protein than FA. The chemical structure of MTFA includes a hydrogenated pteridine ring, rendering it more prone to oxidation [51]. The resultant oxidation products, including 5-methyl-5,6-dihydrofolate and 5-methyltetrahydrofolate pyrazine (Figure 8), display significant oxidative characteristics, potentially functioning as co-oxidants to expedite the oxidation process of SPI [34]. The enhanced oxidizability of these MTFA oxidation products further facilitates the oxidation of sulfhydryl (−SH) groups in SPI [52]. Conversely, SPI oxidation reduces the accumulation of MTFA oxidation products and accelerates the chemical transformation of MTFA into these products.

## 4. Conclusions

This study examined the interactions between three commercial folate compounds—folic acid (FA), L-5-methyltetrahydrofolate (MTFA), and calcium L-5-methyltetrahydrofolate (CMTFA)—and soy protein isolate (SPI) in an aqueous system. We also evaluated how these interactions influenced the structural and physicochemical stability of SPI–folate complexes. Fluorescence-based thermodynamic parameters and simulated molecular docking analyses indicated that both FA and MTFA spontaneously bind to SPI, as evidenced by the negative Gibbs free energy changes (ΔG < 0). The binding of FA to SPI predominantly relied on hydrophobic interactions, accompanied by an increase in entropy (ΔS > 0). In contrast, the binding of MTFA is primarily governed by hydrogen bonding, characterized by a decrease in enthalpy (ΔH < 0). Adding low concentrations of calcium ions (Ca^2+^) in the form of CMTFA did not alter the interaction mechanisms between MTFA and the protein. The experimental results on stability indicated that the formation of SPI–folate complexes enhances both the physical and thermal stability of SPI by stabilizing the protein conformation without altering its structure. The results regarding the chemical stability of folates indicated that the complexes effectively inhibited the degradation of FA induced by light exposure. Conversely, the presence of SPI facilitated the degradation of MTFA through oxidation, which could be ascribed to the synergistic interaction between MTFA and SPI, leading to the generation of endogenous reactive oxygen species. This study creates awareness of the potential of SPI as a carrier for loading various unstable compounds, each exhibiting distinct interaction and degradation mechanisms.

## Figures and Tables

**Table 1:** Quenching constant (K_q_), binding constant (K_a_), and thermodynamic parameters of soy protein isolation–folate complexes at 298.2–308.2 K.

System	T (K)	*K*_q_ (×10^12^ L·mol^−1^·s^−1^)	*K*_a_ (×10^4^ M^−1^)	n	ΔG (kJ·mol^−1^)	ΔH (kJ·mol^−1^)	ΔS (J·mol^−1^K^−1^)
FA	298.2	5.97 ± 0.31	2.72 ± 0.24	1.15 ± 0.05	−31.00 ± 1.37	51.73 ± 1.67	277.65 ± 5.51
	303.2	5.73 ± 0.28	3.90 ± 0.10	1.18 ± 0.02	−32.43 ± 0.58		
	308.2	5.59 ± 0.30	5.35 ± 0.16	1.22 ± 0.03	−33.77 ± 0.94		
MTFA	298.2	4.64 ± 0.22	2.70 ± 0.13	1.17 ± 0.03	−30.99 ± 0.74	−60.13 ± 11.10	−97.44 ± 36.65
	303.2	4.61 ± 0.26	2.08 ± 0.19	1.15 ± 0.04	−30.85 ± 1.04		
	308.2	4.38 ± 0.11	1.45 ± 0.06	1.11 ± 0.01	−30.01 ± 1.71		
CMTFA	298.2	4.40 ± 0.14	2.69 ± 0.11	1.17 ± 0.02	−30.97 ± 0.63	−60.11 ± 6.03	−97.57 ± 19.91
	303.2	4.73 ± 0.17	1.93 ± 0.06	1.13 ± 0.01	−30.66 ± 0.35		
	308.2	4.27 ± 0.23	1.22 ± 0.28	1.10 ± 0.06	−29.99 ± 1.65		

**Table 2:** The particle size and ζ-potential of soy protein isolation (SPI)–folate (FA and MTFA) complexes before and after two different storage conditions.

Condition	SPI		SPI + FA	
	Particle Size (nm)	ζ-Potential (mV)	Particle Size (nm)	ζ-Potential (mV)
0 days	30.6 ± 1.1	−10.3 ± 0.2	30.3 ± 0.9	−10.5 ± 0.3
9 days in light	61.9 ± 2.8	−15.6 ± 0.7	55.7 ± 2.8	−14.4 ± 1.6
60 days in dark	45.0 ± 1.9	−14.2 ± 1.3	34.9 ± 1.0	−15.4 ± 0.5
**Condition**	**SPI + MTFA**		**SPI + CMTFA**	
	**Particle Size (nm)**	**ζ-Potential (mV)**	**Particle Size (nm)**	**ζ-Potential (mV)**
0 days	30.1 ± 0.6	−10.4 ± 0.3	30.3 ± 0.3	−10.6 ± 0.2
3 days in light	41.5 ± 1.2	−14.0 ± 0.4	41.7 ± 2.8	−14.6 ± 0.8
7 days in dark	32.2 ± 0.5	−14.2 ± 0.7	32.1 ± 0.8	−15.0 ± 1.1

## Data Availability

The data presented in this study are available on request from the corresponding author. The data are not publicly available due to privacy restrictions.

## Supplementary Materials

The following supporting information can be downloaded at https://www.mdpi.com/article/10.3390/foods13244033/s1, Figure S1. Van’t Hoff plots for the binding interaction of soy protein isolate with folic acid (FA) and 5-methyltetrahydrofolate (MTFA), as well as MTFA in the presence of 25 µM divalent calcium ion (Ca^2+^). Figure S2. LC-MS report for MTFA in the control samples and the SPI–MTFA complexes after 3 days of light conditions. Figure S3. LC-MS report for MTFA in the control samples and the SPI–MTFA complexes after 7 days of dark conditions. Figure S4. LC-MS report for CMTFA in the control samples and the SPI–MTFA complexes after 3 days of light conditions. Figure S5. LC-MS report for CMTFA in the control samples and the SPI–MTFA complexes after 7 days of dark conditions.
