# Supplementary material for: Comparison of Interactions Between Soy Protein Isolate and Three Folate Molecules: Effect on the Stabilization, Degradation, and Oxidization of Folates and Protein
Source: Foods. 2024 Dec 13;13(24):4033. doi: 10.3390/foods13244033 (PMC11727515; doi:10.3390/foods13244033)
Supplement: Supplementary file 1 [file foods-13-04033-s001.zip › foods-3321370-supplementary.pdf]

Supplementary Materials:

Comparison of Interactions between Soy Protein Isolate and Three Folate Molecules: Effect on the Stabilization, Degradation, and Oxidization of Folates and Protein

Linlin He [1], Yuqian Yan [1], Dandan Song [1], Shuangfeng Li [2], Yanna Zhao [1], Zhuang Ding [1,*], and Zhengping Wang [1]

[1] Institute of Biopharmaceutical Research, Liaocheng University, Liaocheng 252059, China

[2] School of Pharmaceutical Science and Food Engineering, Liaocheng University, Liaocheng 252059, China

* Correspondence: dingzhuang@lcu.edu.cn (Z. D.); Tel./Fax: +86-635-8239136 (Z. D.)

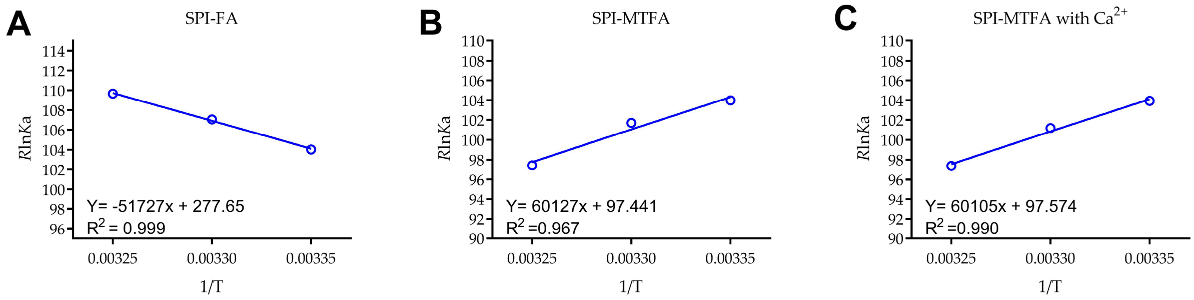

Figure S1. Van't Hoff plots for the binding interaction of soy protein isotion with folic acid (FA) (**A**) and 5-methyltetrahydrofolate (MTFA) (**B**), as well as MTFA under 25 μM divalent calcium ion (Ca^{2+}) (**C**).

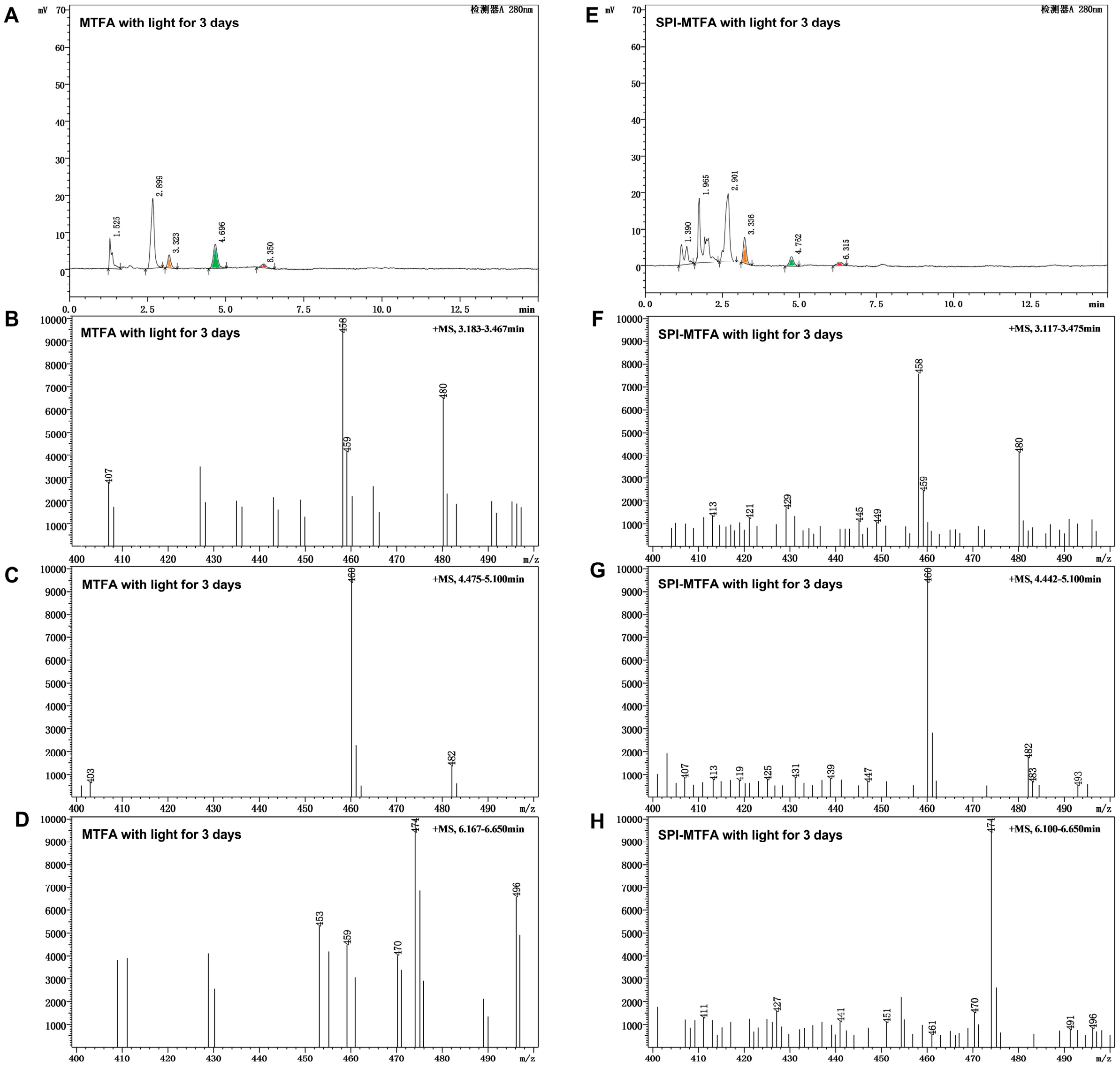

Figure S2. (A-D) LC-MS report of MTFA in the control samples after 3 days of light conditions. Peak appeared in 3.323 min of retention time is oxidation product I (OXP-I) with a 458 m/z [$M+H^+$]; peak appeared in 4.696 min of retention time is MTFA with a 460 m/z [$M+H^+$]; peak appeared in 6.350 min of retention time is oxidation product II (OXP-II) with a 474 m/z [$M+H^+$]. **(E-G)** LC-MS report of MTFA in the SPI-MTFA complexes after 3 days of light conditions. Peak appeared in 3.336 min of retention time is oxidation product I (OXP-I) with a 458 m/z [$M+H^+$]; peak appeared in 4.762 min of retention time is MTFA with a 460 m/z [$M+H^+$]; peak appeared in 6.315 min of retention time is oxidation product II (OXP-II) with a 474 m/z [$M+H^+$].

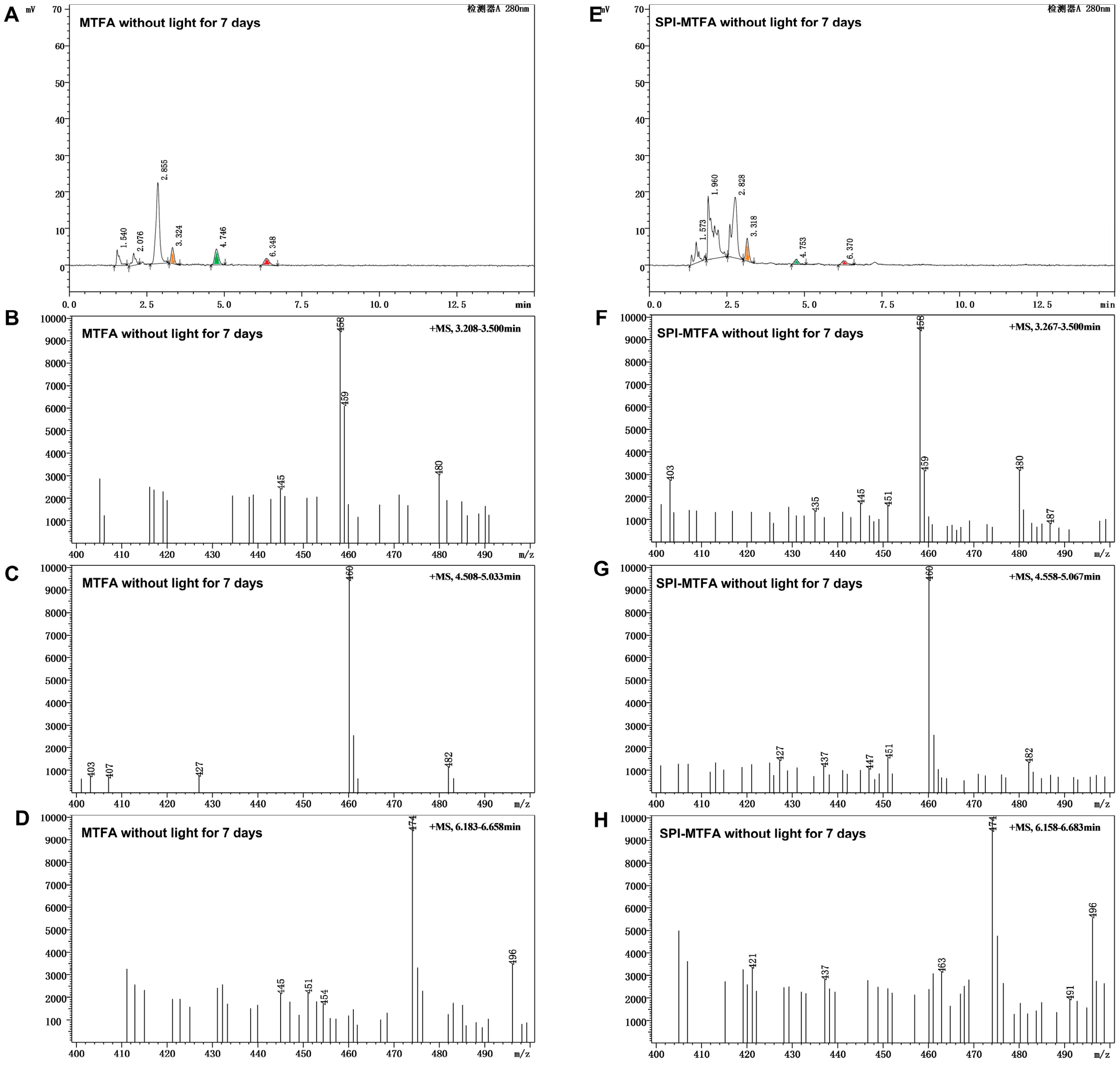

Figure S3. (A-D) LC-MS report of MTFA in the control samples after 7 days of dark conditions. Peak appeared in 3.324 min of retention time is oxidation product I (OXP-I) with a 458 m/z [M+H$^+$]; peak appeared in 4.746 min of retention time is MTFA with a 460 m/z [M+H$^+$]; peak appeared in 6.348 min of retention time is oxidation product II (OXP-II) with a 474 m/z [M+H$^+$]. **(E-G)** LC-MS report of MTFA in the SPI-MTFA complexes after 7 days of dark conditions. Peak appeared in 3.318 min of retention time is oxidation product I (OXP-I) with a 458 m/z [M+H$^+$]; peak appeared in 4.753 min of retention time is MTFA with a 460 m/z [M+H$^+$]; peak appeared in 6.370 min of retention time is oxidation product II (OXP-II) with a 474 m/z [M+H$^+$].

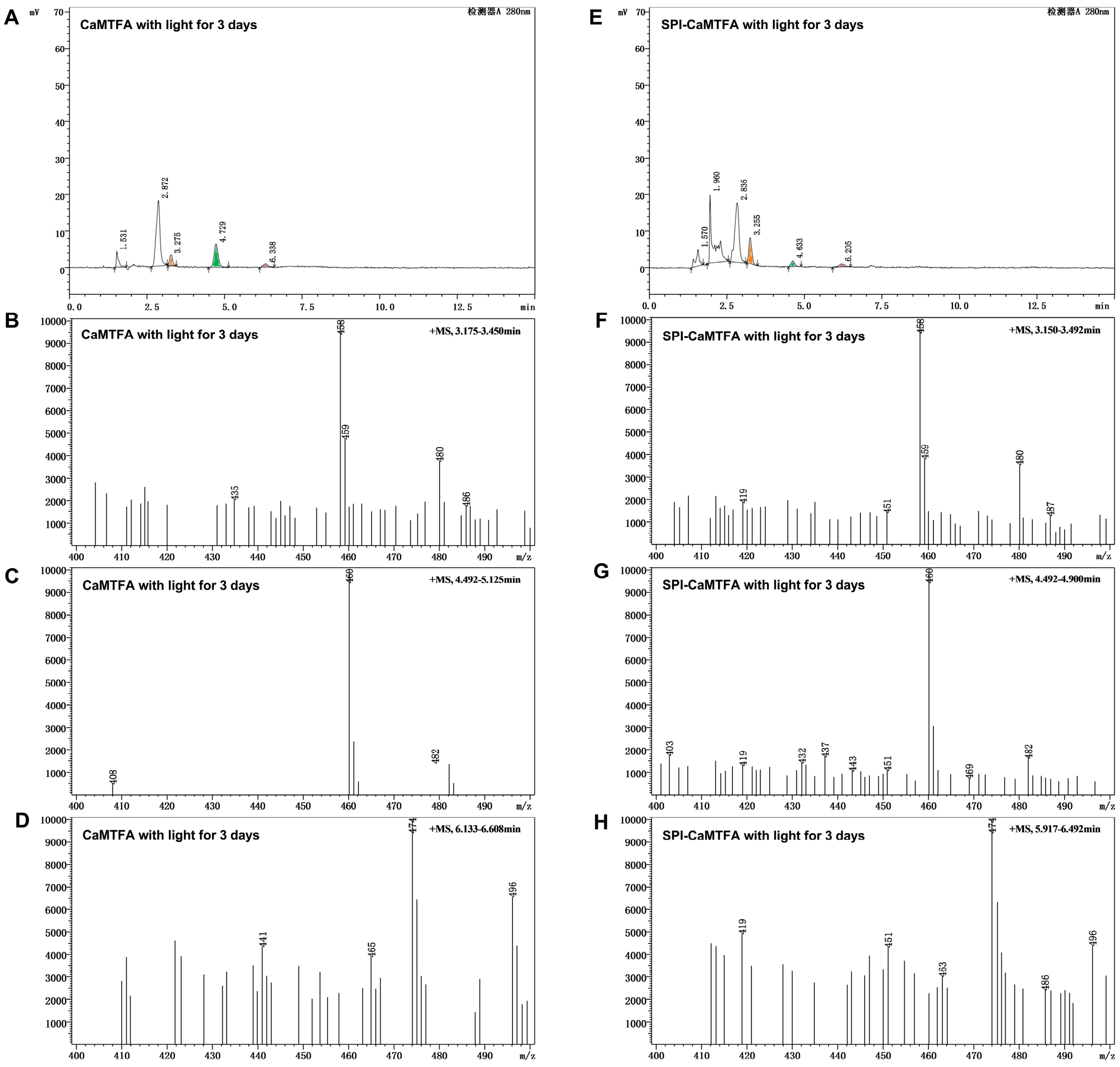

Figure S4. (A-D) LC-MS report of CaMTFA in the control samples after 3 days of light conditions. Peak appeared in 3.275 min of retention time is oxidation product I (OXP-I) with a 458 m/z [M+H$^+$]; peak appeared in 4.729 min of retention time is MTFA with a 460 m/z [M+H$^+$]; peak appeared in 6.338 min of retention time is oxidation product II (OXP-II) with a 474 m/z [M+H$^+$]. **(E-G)** LC-MS report of CaMTFA in the SPI-CaMTFA complexes after 3 days of light conditions. Peak appeared in 3.255 min of retention time is oxidation product I (OXP-I) with a 458 m/z [M+H$^+$]; peak appeared in 4.633 min of retention time is MTFA with a 460 m/z [M+H$^+$]; peak appeared in 6.205 min of retention time is oxidation product II (OXP-II) with a 474 m/z [M+H$^+$].

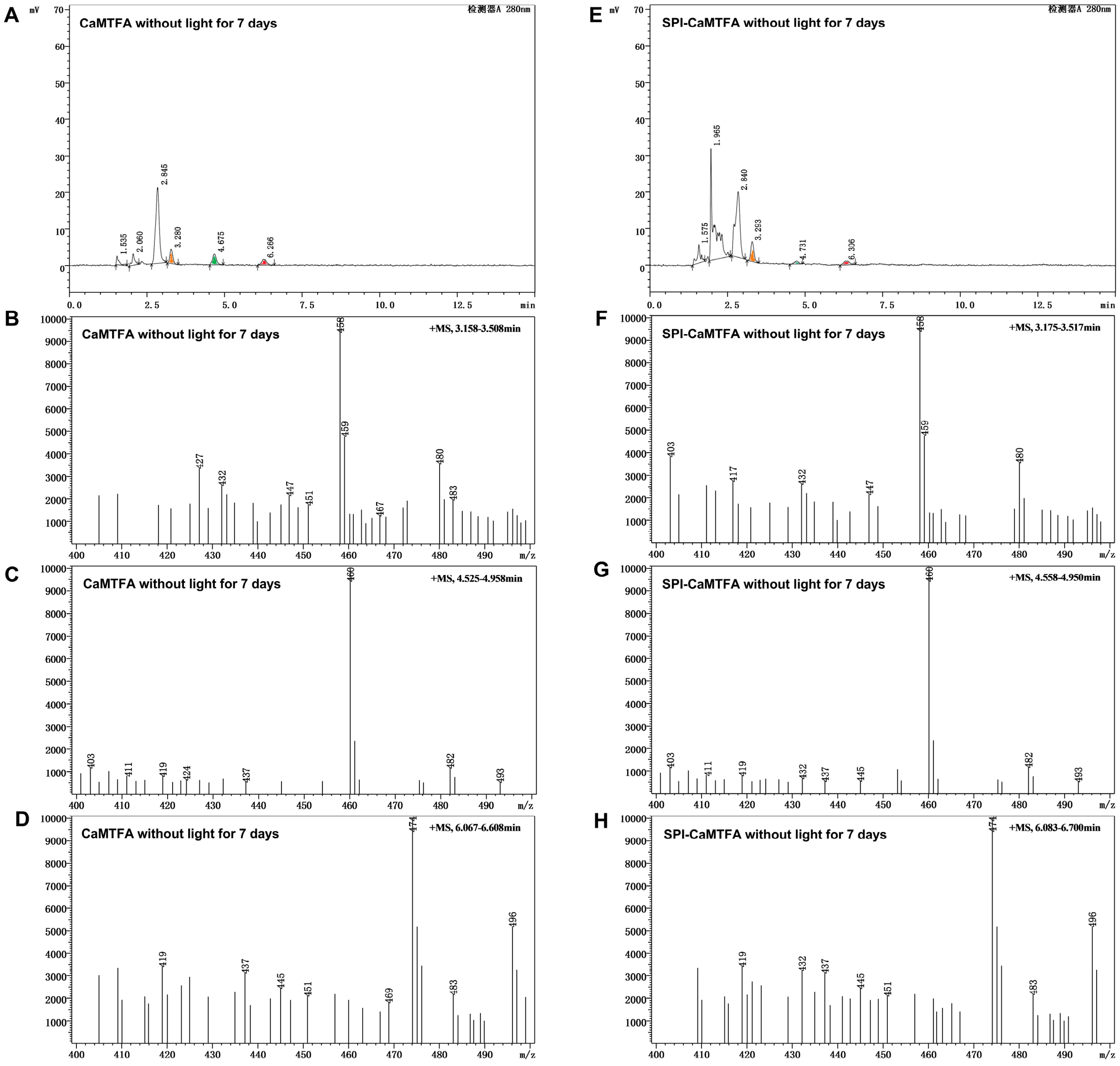

Figure S5. (A-D) LC-MS report of CaMTFA in the control samples after 7 days of dark conditions. Peak appeared in 3.275 min of retention time is oxidation product I (OXP-I) with a 458 m/z [M+H$^+$]; peak appeared in 4.729 min of retention time is MTFA with a 460 m/z [M+H$^+$]; peak appeared in 6.338 min of retention time is oxidation product II (OXP-II) with a 474 m/z [M+H$^+$]. **(E-G)** LC-MS report of CaMTFA in the SPI-CaMTFA complexes after 7 days of dark conditions. Peak appeared in 3.293 min of retention time is oxidation product I (OXP-I) with a 458 m/z [M+H$^+$]; peak appeared in 4.731 min of retention time is MTFA with a 460 m/z [M+H$^+$]; peak appeared in 6.306 min of retention time is oxidation product II (OXP-II) with a 474 m/z [M+H$^+$].
